# Zeroing In on Mindfulness Facets: Similarities, Validity, and Dimensionality across Three Independent Measures

**DOI:** 10.1371/journal.pone.0153073

**Published:** 2016-04-07

**Authors:** Alex B. Siegling, K. V. Petrides

**Affiliations:** London Psychometric Laboratory, University College London, London, United Kingdom; Defence Science and Technology Group, AUSTRALIA

## Abstract

The field of mindfulness has seen a proliferation of psychometric measures, characterised by differences in operationalisation and conceptualisation. To illuminate the scope of, and offer insights into, the diversity apparent in the burgeoning literature, two distinct samples were used to examine the similarities, validity, and dimensionality of mindfulness facets and subscales across three independent measures: the Five Facet Mindfulness Questionnaire (FFMQ), Philadelphia Mindfulness Scale (PHLMS), and Toronto Mindfulness Scale (TMS). Results revealed problematic associations of FFMQ Observe with the other FFMQ facets and supported a four-factor structure (omitting this facet), while disputing the originally envisaged five-factor model; thus, solidifying a pattern in the literature. Results also confirmed the bidimensional nature of the PHLMS and TMS subscales, respectively. A joint Confirmatory Factor Analysis showed that PHLMS Acceptance could be assimilated within the FFMQ’s four-factor model (as a distinct factor). The study offers a way of understanding interrelationships between the available mindfulness scales, so as to help practitioners and researchers make a more informed choice when conceptualising and operationalising mindfulness.

## Introduction

Mindfulness, which can be very broadly understood as living in, and accepting, the present moment non-judgmentally, as opposed to being preoccupied [1–3], has generated a great deal of interest in applied and academic psychology. In applied psychology, it has led new approaches to treating mental illness and developing well-being [4,5]. In academic psychology, the concept has extended beyond its clinical applications to a focus on individual differences. This interest is evident in the recent spurt in psychometric research and proliferation of scales occurring in the past 10 to 15 years. Most of these scales focus on dispositional, or trait, mindfulness (average or baseline states of mindfulness), rather than state mindfulness, or the particular mindful state at the time of measurement [2,6,7]. As described in the next section, eight measures have been salient in the literature [6], although newer ones are emerging [8,9].

Despite overall promising results in terms of criterion and predictive validity[10–14], disagreement spanning the operationalisation and, to a lesser extent, conceptualisation of mindfulness characterises the existing literature, as discussed elsewhere [2,6,15] and described below. Specifically, the existing set of mindfulness scales can be described as heterogeneous, especially in terms of measurement models. The present study examines the similarities, validity, and dimensionality of mindfulness facets and subscales across three independently developed measures of dispositional mindfulness. Throughout the article, our use of terminology is based on the following definitions:

The term *dimension* is used a technical synonym for “construct”; it connotes that a variable (e.g., extraversion) is distinct from other, mostly non-overlapping variables, or dimensions (e.g., neuroticism), rather than being the same construct, or a subfactor thereof. However, a single dimension may still be multi-faceted (as opposed to multidimensional). *Facets* are theoretically derived variables used to ascertain that all relevant content areas are represented in a measure [16]; they are interrelated variables that represent narrow and homogenous subsets of affective, behavioural, or cognitive manifestations (in psychometric terms items) of a given construct. The term *subscale* can be used to refer to any type of scale score of a given measure other than the global composite, including facet and factor scores. However, this term is reserved here for scales that are part of the same measure, but which are not sufficiently interrelated to yield a higher-order factor, in order to distinguish these variables from facets.

### Measures and Facets of Dispositional Mindfulness

The established mindfulness scales differ conceptually and operationally beyond their differentiation as trait versus state measures [15,2,6,7]. The unidimensional facet or item measures organise their facets or items within a hierarchical model, under a single mindfulness factor. While these measures may all represent the same construct [17], they are diverse in terms of their underlying structural models and representations of mindfulness; some of the measures are broader in scope, presumably assessing the construct more comprehensively, whereas others have a narrower focus, measuring only some of its elements.

The Five Facet Mindfulness Questionnaire (FFMQ) [18], the Kentucky Inventory of Mindfulness Skills (KIMS) [19], the Southampton Mindfulness Questionnaire (SMQ) [20], and the Cognitive and Affective Mindfulness Scale-Revised (CAMS–R) [21] comprise either four or five facets that vary between measures. However, only the FFMQ and KIMS have facet scores suitable for use in research and of satisfactory reliability [18,19], while the SMQ and CAMS–R use facets for representational purposes only (that is to say, the content of the facets is represented in a total score, but the measures do not yield facet scores) [20,21]. Another two unidimensional measures, the Mindful Attention Awareness Scale (MAAS) [22] and the Freiburg Mindfulness Inventory (FMI) [23] directly operationalise the general mindfulness factor from their respective items; they do not use facets to represent the construct.

A distinct measure based on a hierarchical model is the Langer Mindfulness Scale [24,25], which is grounded in a somewhat divergent conceptualisation of mindfulness: as “a state in which one is open to novelty, alert to distinctions, sensitive to context, aware of multiple perspectives, and oriented in the present” [25] (p. 1). Its facets are Novelty Seeking, Novelty Producing, and Engagement. The underlying conceptualisation has been described as a Western approach that, despite similarities, differs from the traditional perspectives, which are linked to Eastern religions and provide the basis for the bulk of psychometric measures. More detailed information on differences between conceptualisations can be found in published reviews, such as in [15].

The FFMQ was empirically derived by factor-analysing the items of the other five unidimensional facet or item scales (KIMS, CAMS–R, SMQ, MAAS, and FMI). For this reason, it can be considered a relatively comprehensive operationalisation of the construct that may supersede its constituent scales in terms of breadth and construct validity. The FFMQ consists of five facets (Describe, Act with Awareness, Accept without Judgment, and Nonreact), four of which (not including Nonreact) also constitute its main predecessor, the KIMS [19].

On the other hand, the FFMQ model, and in particular its Observe facet, has produced problematic results. A four-factor hierarchical model omitting the Observe facet tends to results in better model fit for the FFMQ than the originally envisaged five-factor model; increasing evidence supports a five-factor structure including Observe in meditators only [18,19,26–30]. Alternatively, a bidimensional facet model incorporating all five facets under two weakly associated second-order factors has also been identified and partially confirmed for a short form of the FFMQ in both meditators and non-meditators [31,32]. While intercorrelations among the FFMQ facets are generally significant and weak-to-moderate, as one would expect, the Observe facet has often shown non-significant, and sometimes even negative, correlations with one or more of the other four facets, such as Act with Awareness and Accept without Judgment, as well as weak factor loadings [18,19,26,32–34]. In terms of criterion validity, FFMQ Observe was found to buffer the effect of stress in meditators only [35] and to have negligible incremental validity over the other facets in predicting construct-relevant criteria, including some detrimental effects [10,36–38].

A further two measures are grounded in the mainstream conceptualisation of mindfulness, but they diverge operationally in their bidimensional structure, consisting of two subscales that correlate weakly or non-significantly. These are the Toronto Mindfulness Scale (TMS) [39] and the Philadelphia Mindfulness Scale (PHLMS) [40]. The PHLMS was explicitly designed to operationalise two orthogonal subscales, labelled Awareness and Acceptance, which did not correlate (*r* = -.06) [40]. Although the TMS was created to permit oblique factors, its subscale correlations were not large enough to argue that a single shared dimension accounts for much of their variance, and they were only reported for the state version (*r* = .26 to .42) [41]. Thus, its two subscales were interpreted as assessing distinct, but related latent constructs, labelled Curiosity and Decenter. It is important to bear in mind that using heterogeneous measures consisting of weakly related or orthogonal factors to represent a single construct is problematic [42,43]. Although neither of these two measures claims to assess a single global construct, both are linked to the extant literature (i.e., the concept of mindfulness) and depart from the other measures in their bidimensional focus.

Table 1 presents definitions for the FFMQ facets (Describe, Act with Awareness, Accept without Judgment, and Nonreact) and PHLMS and TMS subscales, along with sample items. A triplet of similar facets across the three focal measures consists of FFMQ Observe, PHLMS Awareness, and TMS Curiosity. Despite some differences, all three concern a deliberate perceptual focus on present-moment experiences. A pair of very similar facets consists of FFMQ Accept without Judgment and PHLMS Acceptance, both of which reflect a person’s tendency to accept, rather than judge, internal and external experiences. Another pair of similar facets is that of FFMQ Nonreact and TMS Decenter, both reflecting (emotional) disengagement from one’s inner feelings, perceptions, and thoughts.

**Table 1:** Operationalisation of Mindfulness across Multi-Faceted Measures, Including Facet or Subscale Definitions and Sample Items.

Measure	Scales	Definition	Sample item
**FFMQ**	Observe*	Tendency to observe, notice, or attend to internal and external phenomena.	I intentionally stay aware of my feelings.
	**Describe**	Tendency to Describe or label sensations, perceptions, thoughts, emotions, etc. with words.	My natural tendency is to put my experiences into words.
	**Act with Awareness**	Tendency to focus undivided attention on the current activity or avoiding automatic pilot; concentration.	I easily get lost in my thoughts and feelings.
	Accept w/o Judgment**	Tendency to accept without making judgments or evaluations.	I disapprove of myself when I have irrational ideas.
	Nonreact***	Tendency not to react to one’s experience.	I watch my feelings without getting lost in them.
**PHLMS**	Awareness*	Tendency to be highly aware of one’s internal and external experiences.	When I am startled, I notice what is going on inside my body.
	Acceptance**	Tendency to accept and not to judge internal and external experiences.	I try to put my problems out of mind.
**TMS**	Curiosity*	Stance of wanting to learn more about one’s experiences.	I am curious about each of my thoughts and feelings as they occur.
	Decenter***	Tendency to relate to one’s thoughts or feelings in a wider field of Awareness rather than being overly absorbed in one’s internal experiences.	I experience myself as separate from my changing thoughts and feelings.

Conceptually similar facets are denoted by the number of asterisks. FFMQ = Five Facet Mindfulness Questionnaire [18]; PHLMS = Philadelphia Mindfulness Scale [40]; TMS = Toronto Mindfulness Scale [39].

### Convergent Validity

Research has shown that, aside from the LMS, the unidimensional facet or item measures show correlations of mostly moderate strength [18,20,21], which speaks to the differences in how the construct is operationalised across these measures. In a previous study, [17] wondered whether these less than satisfactory levels of convergence are a function of primarily different constructs being assessed between measures (multidimensionality), as opposed to differences in the extent to which these measures operationalise the same construct (construct representation). A single component was found to underlie the shared variance among these scales, all of which loaded well on it. Thus, it appears that they all tap into the same construct, but vary in terms of breadth and focus. On the other hand, the conceptually distinct Langer Mindfulness Scale loaded relatively weakly on this dimension and much stronger on an orthogonal, second dimension, suggesting that it primarily measures a distinct construct [17]. This inference is further supported by the scale’s relatively weak associations with the other measures [17,24].

Of the two bidimensional measures, the PHLMS subscales were found to correlate weakly to moderately with the MAAS in non-clinical and psychiatric samples [40]. Correlations with facet scores of a multi-faceted measure, the KIMS, were mostly in line with conceptual similarities: PHLMS Awareness correlated strongly with the KIMS Observe facet and PHLMS Acceptance had the strongest correlation with the KIMS Accept without Judgment facet. Both subscales of the TMS (trait version) were associated with the unidimensional facet or item scales, ranging from weak to moderate for TMS Curiosity and from moderate to strong for TMS Decenter [39]. At the facet level of the FFMQ and KIMS [39], TMS Curiosity had to strongest correlation with FFMQ/KIMS Observe, followed by FFMQ Nonreact (both within a moderate range); its correlations with the remaining facets were modest in strength. TMS Decenter was most highly related to FFMQ Nonreact, moderately with most other KIMS/FFMQ facets (Observe, Act with Awareness, and Accept without Judgment), and weakly with the Describe facet. Again, these correlations support the conceptual similarities between the facets and subscales of these measures.

Overall, there has been little empirical effort to systematically examine facet (or subscale) interrelationships and similarities among the key independently developed measures (FFMQ, PHLMS, and TMS) and to establish if all facets and subscales represent elements of the mainstream conceptualisation of mindfulness, as assessed by the bulk of scales. A related specific concern is whether the FFMQ Observe facet, and possibly the conceptually and empirically related PHLMS Awareness and TMS Curiosity subscales, represent valid elements of this construct.

### The Present Study

The purpose of this study was to shed light on the similarity, validity, and dimensionality among independently measures of dispositional mindfulness, especially their facets or subscales (additional measures were used for validation purposes). The internal (factorial) structure of these measures was examined in the study samples, either by means of associations (PHLMS and TMS) or by testing competing structural models (FFMQ). Joint Exploratory and Confirmatory Factor Analysis examined which facets and subscales share a common dimension (mindfulness) and how their variance can be summarised in an optimal way. Of related interest was if any of the PHLMS and TMS subscales converge with conceptually similar FFMQ facets, or if these measures mostly tap into distinct constructs.

## Method

### Participants

This research was approved by the UCL Research Ethics Committee (Project ID Number: CEHP/2010/013).

The two samples of this study were previously used for different purposes in [17]. Sample 1 (*N* = 395) consisted mainly of students at a British university, but also included other individuals affiliated with the same institution. Participants were recruited via the institutional subject pool over approximately one year (February 2012–March 2014). Most of them were female (76.7%) and ages ranged from 18 to 57 years (*M* = 21.9, *SD* = 4.9). They were of White (55.4%), East Asian (28.4%), South Asian (Indian, Pakistani, and Bangladeshi [8.8%]), and a few other (8.4%) backgrounds. All participants were entered into a prize draw for gift cards and most received course credit for their participation.

Sample 2 (*N* = 172) was recruited online via recruitment platforms for psychological research or social media. Where social media were utilised, two authors and public promoters of mindfulness kindly posted a recruitment notice for the study. This sample had an average age of 36.9 years (*SD* = 14.1) and a broad age range of 18 to 76 years, both markedly higher than in Sample 1. However, a similar proportion of the participants (79.7%) were female. In terms of ethnic backgrounds, the sample was fairly homogenous: 84.3% Caucasian, 2.9% East Asian, 1.7% South Asian, 4.7% Black, and 6.4% other/mixed. As a token of appreciation, all participants were entered into a price draw for gift cards.

These descriptions concern the valid cases only, whereas “bad cases”, such as drop outs, were removed prior to data analysis from each sample. Specifically, participants who had completed the respective measures partially or skipped numerous items, presumably due to reasons other than any plausible discomfort caused by the items, were deleted from the dataset. Isolated missing items were deemed acceptable. Those who did not take the survey seriously (e.g., giving the same responses throughout or using offensive language in the demographics section) or had unrealistically fast completion times were also removed.

### Measures and Procedure

After giving electronic consent by ticking of the relevant box, both samples completed the following mindfulness scales via an anonymous online survey system: FFMQ [18], PHLMS [40], TMS (trait version) [39], KIMS [19], CAMS–R) [21], SMQ [20], MAAS [22], and FMI [23]. Data for the MAAS and FMI were only available for 115 of the Sample 2 participants.

All scales are based on self-report, using a Likert scale response format with 4, 5, 6, or 7 points. Properties of these measures, including number of items and internal consistencies in the two study samples, are shown in Table 2. As shown, the levels of internal reliability range from acceptable to strong in both samples and across measures.

**Table 2:** Descriptive Statistics and Properties of Mindfulness Scales.

Scales	No. of items	Sample 1 (*N* = 395)					Sample 2 (*N* = 172)				
		α	*M*	*SD*	Skewness	Kurtosis	α	*M*	*SD*	Skewness	Kurtosis
**FFMQ Observe**	8	.80	3.21	0.69	0.13	-0.13	.82	3.48	0.66	-0.31	0.51
**FFMQ Describe**	8	.88	3.26	0.74	-0.01	-0.42	.93	3.39	0.90	-0.27	-0.35
**FFMQ Act with Awareness**	8	.88	3.10	0.73	-0.08	0.16	.91	2.95	0.77	0.10	-0.24
**FFMQ Accept w/o Judgment**	8	.91	3.00	0.85	-0.11	-0.31	.94	2.87	0.99	0.22	-0.60
**FFMQ Nonreact**	7	.83	2.86	0.68	0.22	0.10	.88	2.70	0.72	0.11	-0.14
**PHLMS Awareness**	10	.78	3.51	0.55	-0.06	0.06	.85	3.69	0.62	-0.44	0.47
**PHLMS Acceptance**	10	.82	2.71	0.64	-0.08	-0.16	.88	2.71	0.76	0.05	-0.59
**TMS Curiosity**	6	.86	2.50	0.80	-0.32	-0.12	.88	2.51	0.83	-0.41	-0.20
**TMS Decenter**	7	.73	1.87	0.67	-0.01	-0.07	.77	1.71	0.73	0.00	-0.37
**KIMS**	39	.81	3.06	0.35	0.25	0.91	.89	3.13	0.46	0.01	0.76
**CAMS–R**	12	.75	2.55	0.43	-0.08	0.09	.83	2.46	0.50	0.21	-0.32
**SMQ**	16	.80	3.25	0.74	-0.36	0.06	.87	3.02	0.91	-0.11	0.05
**MAAS**	15	.86	3.67	0.72	0.11	0.19	.88	3.60	0.80	-0.20	0.06
**FMI**	14	.83	2.57	0.47	0.01	0.32	.89	2.40	0.59	0.16	-0.39

Of the Sample 2 participants, only 115 completed the MAAS and FMI. FFMQ = Five Facet Mindfulness Questionnaire [18]; PHLMS = Philadelphia Mindfulness Scale [40]; TMS = Toronto Mindfulness Scale [39]; KIMS = Kentucky Inventory of Mindfulness Skills [19]; CAMS–R = Cognitive and Affective Mindfulness Scale–Revised [21]; SMQ = Southampton Mindfulness Questionnaire [20]; MAAS = Mindful Attention Awareness Scale [40]; FMI = Freiburg Mindfulness Inventory [23].

To balance the effects of any extraneous factors, such as testing fatigue, the scales were administered in randomised order and the order of items within each scale was also randomised across participants. Upon submitting their responses on each scale, participants were automatically notified of any missing responses and given the opportunity to add them.

### Statistical Analysis

Intercorrelations among the PHLMS and TMS subscales and FFMQ facets were examined to assess the between-scale levels of similarity and intra-scale relations and structure. Next, bivariate correlations of PHLMS and TMS subscales with mindfulness, a comprehensive mindfulness component extracted from the unidimensional facet or item scales (KIMS, CAMS–R, SMQ, MAAS, and FMI), were examined to assess the extent to which these subscales map onto the mainstream conceptualisation of mindfulness. The component extracted from these measures was considered to give a more valid representation than the FFMQ alone (this is due to the uncertain structure of the FFMQ, in which individual facets, including their specific variances, carry greater weight). The FFMQ, which derives from these five measures, was not included in this composite so its content would not be overrepresented.

Confirmatory Factor Analysis was used to compare the five- and four-factor hierarchical models of the FFMQ (the respective sample sizes can be considered sufficient for this purpose, given numbers of parameters; [44], as well as to examine which PHLMS and TMS subscales within the better-supported FFMQ model; thus, representing the same construct. These analyses were conducted on item parcels, as executed in the construction of the FFMQ [18] and in later tests of its factor structure [30]. Specifically, three parcels per facet were created, by assigning the items in the order in which they appear in the FFMQ across parcels (e.g., Describe Item 1 → Describe Parcel 1, Describe Item 2 → Describe Parcel 2, and so forth). Since justifications for the use of item parcels in this context were previously presented in [18], they will not be reiterated here.

Whereas the two FFMQ models were tested via Confirmatory Factor Analysis in both samples, the combined structure of all three measures was first examined in a joint Exploratory Factor Analysis in Sample 2 (the smaller sample) and subsequently tested via Confirmatory Factor Analysis in Sample 1. Exploratory Factor Analysis was executed using principal-axis factoring method of extraction, with oblique rotation (Promax method, delta = 4); Confirmatory Factor Analysis used maximum-likelihood estimation, whereby model fit was determined using the following indices: Goodness of Fit Index (GFI), Comparative Fit Index (CFI), Normed Fit Index (NFI), Root Mean Square Error of Approximation (RMSEA), and Standardised Root Mean Square Residual (SRMR). In line with current thinking on adequate mode fit [45], the criteria for the various fit indices were: GFI ≥ .95, CFI ≥ .95, NFI ≥ .95, RMSEA ≤ .07, and SRMR ≤ .08.

## Results

Although rare, especially after data cleaning, missing responses were compensated by using mean item scores for each facet or subscale. Scatter plots, along with more objective indices of normality included in Table 2, were examined and taken as evidence for normality.

### Mindfulness Component Extraction

Principal Component Analysis results for the five mindfulness scales are shown in Table 3. A scree plot, Kaiser’s criterion (of Eigenvalues greater than 1), and parallel analysis unanimously showed that a single principal component was accountable for the shared variance of the five scales. Specifically, the component explained 56.6% and 67.5% of the scales’ shared variance in Samples 1 and 2, respectively. Component loadings of the scales ranged from .64 (MAAS, Sample 1) to .87 (CAMS-R, Sample 2). The derived factor score was used in subsequent analyses, where it is referred to as “mindfulness component”.

**Table 3:** Principal Component Analysis of Mindfulness Scales.

Sample	Scale	Factor loading	Communality	% of variance
**1**	**KIMS**	.78	.61	56.59
	**CAMS–R**	.68	.46	
	**SMQ**	.64	.41	
	**MAAS**	.78	.62	
	**FMI**	.86	.73	
**2**	**KIMS**	.86	.73	67.53
	**CAMS–R**	.80	.64	
	**SMQ**	.68	.46	
	**MAAS**	.88	.77	
	**FMI**	.87	.76	

*N* = 395 for Sample 1 and 115 for Sample 2. KIMS = Kentucky Inventory of Mindfulness Skills [19]; CAMS–R = Cognitive and Affective Mindfulness Scale–Revised [21]; SMQ = Southampton Mindfulness Questionnaire [20]; MAAS = Mindful Attention Awareness Scale [22]; FMI = Freiburg Mindfulness Inventory [23].

### Correlations

Zero-order correlations for the FFMQ facets, PHLMS and TMS subscales, and mindfulness component are shown in Table 4. Strengths of significant associations among these scores appear to be generally stronger in Sample 2. Concerning the FFMQ, facets were mostly non-significantly or weakly associated in Sample 1 and weakly to moderately in Sample 2. Of note, the Observe facet showed significant negative correlations with Act with Awareness and Accept without Judgment, whereas the same correlations were non-significant in Sample 2.

**Table 4:** Intercorrelations among FFMQ Facets, PHFLMS and TMS Subscales, and the Global Mindfulness Component.

Sample	Scale	Mindfulness	1	2	3	4	5	6	7	8
**1**	**1. FFMQ Observe**	—								
** **	**2. FFMQ Describe**	—	.23***							
** **	**3. FFMQ Act with Awareness**	—	-.11*	.08						
** **	**4. FFMQ Accept w/o Judgment**	—	-.22***	.09	.44***					
** **	**5. FFMQ Nonreact**	—	.23***	.07	.09	.13*				
** **	**6. PHLMS Awareness**	.26***	.62***	.40***	.04	-.11*	.09			
** **	**7. PHLMS Acceptance**	.41***	-.20***	.07	.39***	.58***	.16**	-.28***		
** **	**8. TMS Curiosity**	.12*	.40***	.17***	-.04	-.21***	.10*	.42***	-.14**	
** **	**9. TMS Decenter**	.38***	.33***	.06	.02	-.02	.51***	.24***	.00	.39***
**2**	**1. FFMQ Observe**	—								
** **	**2. FFMQ Describe**	—	.31***							
** **	**3. FFMQ Act with Awareness**	—	.13	.30***						
** **	**4. FFMQ Accept w/o Judgment**	—	.05	.21**	.56***					
** **	**5. FFMQ Nonreact**	—	.36***	.17*	.27***	.45***				
** **	**6. PHLMS Awareness**	.42***	.67***	.41***	.17*	.04	.25***			
** **	**7. PHLMS Acceptance**	.63***	.06	.25***	.53***	.72***	.46***	-.04		
** **	**8. TMS Curiosity**	.24*	.35***	.24**	-.03	-.03	.24**	.30***	.07	
** **	**9. TMS Decenter**	.43***	.29***	.14	.12	.26***	.55***	.14	.34***	.40***

*N* = 395 for Sample 1 and 172 for Sample 2. Global mindfulness correlations in Sample 2 are based on the data of 115 participants, who completed all of the mindfulness measures in that sample. FFMQ = Five Facet Mindfulness Questionnaire [18]; PHLMS = Philadelphia Mindfulness Scale [40]; TMS = Toronto Mindfulness Scale [39].

*p < .05.

**p < .01.

***p < .001.

The PHLMS subscales were inversely related in both samples, although coefficients were small and only significant in Sample 1 at *r* = -.28. PHLMS Awareness correlated moderately with the FFMQ Observe and Describe facets in both samples. Its association with the other facets were non-significant or negative (*r* = -.11) in Sample 1 and weak or non-significant in Sample 2. In both samples, the strongest FFMQ correlate of PHLMS Acceptance was Accept without Judgment at *r* = .58 and .72. PHLMS Acceptance also correlated moderately with FFMQ Act with Awareness and weakly to moderately with FFMQ Nonreact in both samples. Its association with FFMQ Describe was non-significant in Sample 1 and weak in Sample 2. Moreover, the association between PHLMS Acceptance and FFMQ Observe was negative in Sample 1 and non-significant in Sample 2.

The two TMS subscales correlated moderately in both samples (*r* = .39 and .40). TMS Curiosity also correlated moderately with FFMQ Observe and weakly with the Describe and Nonreact facets in both samples. Its associations with the other two FFMQ facets were negative and significant in only one instance (a negative correlation with Accept without Judgment in Sample 1). TMS Curiosity correlated moderately with PHLMS Awareness in both samples and either negatively or non-significantly with PHLMS Acceptance. In contrast, TMS Decenter correlated most strongly with FFMQ Nonreact (*r* = .51 and .55), followed by FFMQ Observe (*r* = .33 and .29) in both samples. FFMQ Describe and Act with Awareness were both unrelated to TMS Decenter, whereas Accept without Judgment correlated weakly in Sample 2. Correlations of TMS Decenter were weak with PHLMS Awareness and significant in Sample 2 for PHLMS Acceptance (*r* = .32).

Correlations of the PHLMS and TMS subscales with mindfulness were consistently significant and positive. Most correlations were within a moderate range, but TMS Curiosity and PHLMS Awareness were weaker correlates of this component, although the association for PHLMS Awareness in Sample 2 was also of moderate degree.

### Confirmatory Factor Analysis of FFMQ Hierarchical Models

The five-factor hierarchical model did not fit the data particularly well in either Sample 1, χ^2^(85) = 216.99, *p* < .001, CFI = .96, GFI = .93, NFI = .94, RMSEA = .06, SRMR = .08, or Sample 2, χ^2^(85) = 170.37, *p* < .001, CFI = .95, GFI = .89, NFI = .91, RMSEA = .08, SRMR = .10. Also, the FFMQ Observe loaded negatively on the latent mindfulness factor in Sample 1 (λ = -.17, *p* = .03), whereas in Sample 2, its loading on mindfulness was relatively small (λ = .22, *p* = .02). In contrast, the four-factor hierarchical model without the Observe facet fit the data very well in both Sample 1, χ^2^(50) = 88.51, *p* = .001, CFI = .99, GFI = .96, NFI = .97, RMSEA = .04, SRMR = .04, and Sample 2, χ^2^(50) = 61.28, *p* = .13, CFI = .95, GFI = .99, NFI = .96, RMSEA = .04, SRMR = .06.

**Fig 1:**
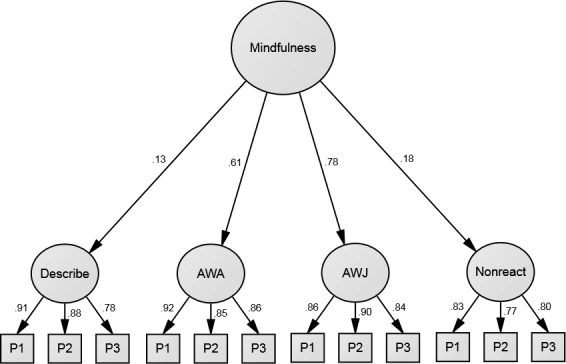
Confirmatory Factor Analysis results for the four-factor hierarchical model of the Five Facet Mindfulness Questionnaire [18], omitting the Observe facet, in Sample 1 (*N* = 395). First-order latent variables represent the four facets and derive from item parcels (three per facet). Error terms are omitted for visual clarity. AWA = Act with Awareness; AWJ = Accept w/o Judgment; P1 to P3 = Parcels 1 to 3. All standardised coefficients are significant at the .05 level, with the exception of the path from Mindfulness to Describe, which did not reach significance (*p* = .09).

**Fig 2:**
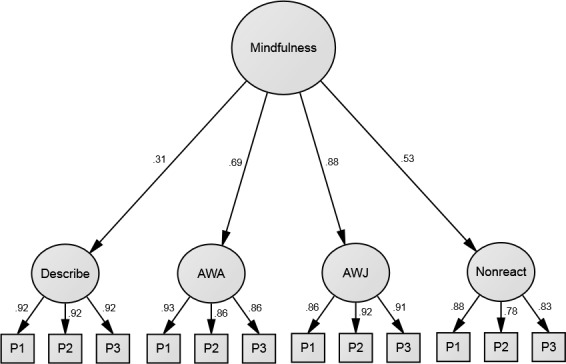
Confirmatory Factor Analysis results for the four-factor hierarchical model of the Five Facet Mindfulness Questionnaire [18], omitting the Observe facet, in Sample 2 (*N* = 172). First-order latent variables represent the four facets and derive from item parcels (three per facet). Error terms are omitted for visual clarity. AWA = Act with Awareness; AWJ = Accept w/o Judgment; P1 to P3 = Parcels 1 to 3. All standardised coefficients are significant at the .01 level.

Factor loadings for the better supported four-factor model are shown in Figs 1 and 2, pertaining to Samples 1 and 2, respectively. Although this model fit the data well in both samples, magnitudes of standardised path coefficients were heterogeneous at the facet level. Specifically, facet loadings were low for Describe in both samples (especially in Sample 1, where it was not significant) and for Nonreact in Sample 1; they were relatively high for Act with Awareness and Accept without Judgment in both samples.

### Exploratory Factor Analysis of FFMQ, PHLMS, and TMS

Given the preceding results, which fit the consistent pattern in the literature pertaining to the general (non-meditating) population, item parcels representing FFMQ Observe were omitted from the joint factor analysis. A scree plot indicated six to eight factors, while Kaiser’s criterion (Eigenvalues > 1) supported a six-factor solution for the remaining FFMQ parcels when combined with the PHLMS and TMS parcels. Indeed, six clean factors are apparent from the pattern matrix shown in Table 5, with any loadings below the conventional .30 cut-off suppressed; no cross-loadings above .30 appeared. Half the facets and subscales emerged as a distinct factor, characterised by loadings of all three respective item parcels: FFMQ Describe, FFMQ Act with Awareness, TMS Curiosity, and PHLMS Awareness. The two other factors were combinations of (a) FFMQ Accept without Judgment and PHLMS Acceptance, and (b) FFMQ Nonreact and TMS Decenter; each factor was identified by six respective parcels. The solution explained much of the variance in the parcels (76.95%).

**Table 5:** Pattern Matrix for Promax Six-Factor Solution Extracted from FFMQ, TMS, and PHLMS Item Parcels Corresponding to Each Facet or Subscale and Factor Correlation Matrix in Sample 2.Parcel.

	Factor loading					
	1	2	3	4	5	6
FFMQ AWJ P2	1.00					
FFMQ AWJ P3	.88					
FFMQ AWJ P1	.84					
PHLMS Acceptance P2	.70					
PHLMS Acceptance P1	.56					
PHLMS Acceptance P3	.52					
FFMQ Nonreact P1		.83				
FFMQ Nonreact P3		.81				
FFMQ Nonreact P2		.76				
TMS Decenter P3		.59				
TMS Decenter P2		.51				
TMS Decenter P1		.49				
FFMQ Describe P1			.90			
FFMQ Describe P2			.86			
FFMQ Describe P3			.83			
TMS Curiosity P3				.86		
TMS Curiosity P2				.85		
TMS Curiosity P1				.76		
FFMQ AWA P1					.91	
FFMQ AWA P3					.88	
FFMQ AWA P2					.83	
PHLMS Awareness P1						.72
PHLMS Awareness P3						.68
PHLMS Awareness P2						.68
**Eigenvalue**	5.67	4.44	2.86	1.76	1.57	1.18
**% of variance**	23.61	18.52	11.92	7.35	6.53	4.92
	**Factor correlations**					
Factor 1	—					
Factor 2	.29	—				
Factor 3	.21	.13	—			
Factor 4	-.13	.29	.20	—		
Factor 5	.55	.17	.19	-.03	—	
Factor 6	-.19	.10	.32	.30	.00	—

*N* = 172. Factor loadings of < .30 are omitted from the table. FFMQ = Five Facet Mindfulness Questionnaire [18]; PHLMS = Philadelphia Mindfulness Scale [40]; TMS = Toronto Mindfulness Scale [39]; AWJ = Accept w/o Judgment; AWA = Act with Awareness; P1 to P3 = Parcels 1 to 3.

On the other hand, a parallel analysis indicated an eight-factor solution. With the number of factors fixed to eight, every facet or subscale (eight in total) emerged as a distinct factor, characterised by loadings of the respective parcels. The output for this analysis is included in S1 Table. For this reason, it made sense to examine two slightly different models via Confirmatory Factor Analysis in Sample 1; one comprised of six (Model A), and the other of eight (Model B), first-order factors between the item parcels and the second-order, global mindfulness factor. Of note, both models comprise the item parcels of all eight facets or subscales, but in Model A, four of them emerged in pairs as two factors (see Table 5).

### Confirmatory Factor Analysis of FFMQ, PHLMS, and TMS

Initially, both models did not fit the data (Model A: χ^2^[246] = 984.95, *p* < .001, CFI = .86, GFI = .81, NFI = .82, SRMR = .12; Model B: χ^2^[244] = 759.57, *p* < .001, CFI = .90, GFI = .86, NFI = .86, SRMR = .13) and contained two first-order factors that loaded negatively on the global mindfulness factor: PHLMS Awareness and TMS Curiosity. Upon removing these two latent variables and their constituent parcels from each model, Model B yielded satisfactory fit, χ^2^(129) = 326.10, *p* < .001, CFI = .95, GFI = .92, NFI = .92, SRMR = .09, whereas Model A generally did not meet the specified criteria for model fit, χ^2^(131) = 556.48, *p* < .001, CFI = .89, GFI = .85, NFI = .86, SRMR = .07. Moreover, a qui-square difference test showed that Model B fit the data significantly better than Model A, Δχ^2^(2) = 230.38, *p* < .001.

**Fig 3:**
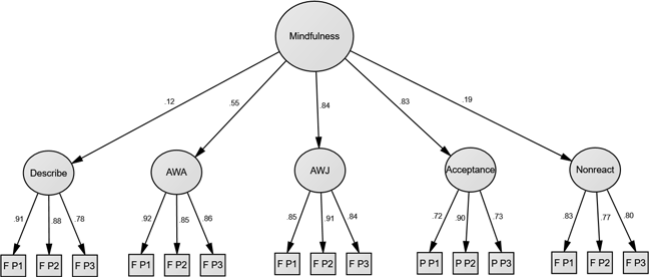
Results for Joint Confirmatory Factor Analysis of the Five Facet Mindfulness Questionnaire (minus the Observe facet) [18], Philadelphia Mindfulness Scale [40], and Toronto Mindfulness Scale [39] in Sample 1 (*N* = 395). First-order latent variables derive from item parcels (three per facet). Error terms are omitted for visual clarity. AWA = Act with Awareness; AWJ = Accept w/o Judgment; F = Five Facet Mindfulness Questionnaire; P = Philadelphia Mindfulness Scale; P1 to P3 = Parcels 1 to 3. All standardised coefficients are significant at the .05 level.

In Model B, in which all remaining facets or subscales represent a distinct second-order factor, the loading for TMS Decenter was non-significant (λ = .04, *p* = .49). Thus, Fig 3 shows a final model with TMS Decenter removed (and without any error covariances added), yielding very good fit, χ2(85) = 161.43, *p* < .001, CFI = .98, GFI = .95, NFI = .96, SRMR = .04. Loadings of the four FFMQ facets were largely unaffected by the additional PHLMS parcels in terms of magnitude with the PHLMS Acceptance factor included in the model. Of note, the loading for FFMQ Describe became significant in this instance. The PHLMS Acceptance subscale loaded highly on mindfulness (.83), consistent with the high loading of the conceptually very similar FFMQ facet (Accept without Judgment).

## Discussion

This study used two distinct samples of English-speaking adults to investigate the similarities, validity, and dimensionality of the PHLMS and TMS subscales, and FFMQ facets. Associations among facets or subscales within measures were examined to verify previously reported relationships. Correlations among the five FFMQ facets included an atheoretical pattern of associations between the Observe facet and two other facets (Act with Awareness and Accept without Judgment), which were negative in Sample 1 and non-significant in Sample 2. These results fit the general pattern of non-significant or even negative associations between FFMQ Observe and some of the other FFMQ facets seen in the literature [18,19,26,32–34]. They are also in line with the non-significant or negative loadings of this facet on the latent mindfulness factor observed here and in previous research [18,19,26–30].

The PHLMS and TMS depart from the bulk of measures in their bidimensional focus, each assessing two relatively narrow and mainly distinct constructs. Whereas the two TMS scores were moderately associated in both samples, the two PHLMS scales were non-significantly associated in one sample, while correlating negatively in the other. For the most part, these results are also in line with previous findings. The subscales of the state version of the TMS correlated weakly to moderately (*r* = .26 to .42) [41] and, accordingly, were interpreted as assessing distinct, but related, latent constructs. In contrast, the PHLMS subscales were explicitly created to be orthogonal, resulting in a non-significant correlation (*r* = -.06) [40]. The significant negative correlation observed here in one sample for the PHLMS subscales can be theoretically accounted for: Awareness is conceptualised as a deliberate behavioural process that directs one’s attention towards a restricted range of experience and, simultaneously, prevents one from being open to, and accepting of, the full range of the psychological experience [40].

The next step taken in the current study was to examine the relationships between the three measures, which partially aimed at assessing the convergent validity of similar facets. PHLMS Awareness correlated with FFMQ Observe and Describe, whereas PHLMS Acceptance correlated with FFMQ Act with Awareness, Accept without Judgment, and Nonreact in both samples. These distinct patterns of associations with mindfulness facets are consistent with the orthogonal nature of the two PHLMS subscales and previously reported correlations with the KIMS facets [40], all of which are also measured with the FFMQ. As can be expected based on conceptual similarity and previous findings, PHLMS Acceptance had the highest associations with FFMQ Accept without Judgment, whereas PHLMS Awareness correlated most strongly with FFMQ Observe.

Both TMS subscales were associated with the FFMQ Observe and Nonreact facets, and TMS Curiosity also correlated with FFMQ Describe. Also in accordance with conceptual similarity and previous findings, FFMQ Observe was the strongest correlate of TMS Curiosity, while FFMQ Nonreact was the strongest correlate of TMS Decenter [39]. Yet, deviating from previously reported associations (Curiosity: *r* = .20, 95% CI = .11 to .29; Decenter: *r* = .43, 95% CI = .35 to .50) [39], both TMS subscales were unrelated to FFMQ Act with Awareness, and TMS Decenter was also unrelated to FFMQ Describe. Furthermore, TMS Curiosity was unrelated to the FFMQ Accept without Judgment in Sample 2 and even correlated negatively with it in Sample 1. The associations reported in the current study better illustrate the bidimensional nature and distinct conceptual meanings of the TMS subscales. Also in line with conceptual resemblance, TMS Curiosity and PHLMS Awareness correlated moderately in both samples.

Associations with a comprehensive mindfulness component previously shown to underlie the shared variance of the unidimensional facet or item measures [17] were examined to assess which of the PHLMS and TMS subscales are valid indicators of the mainstream operationalisation of the construct. These correlations were significant in both samples. The majority of them were within, and none above, a moderate range of .30 to .70, substantiating their conceptualisation as narrower segments of mindfulness. However, correlations for TMS Curiosity and partially PHLMS Awareness were weak, suggesting that they share relatively little variance with mindfulness. Similarly, both TMS subscales previously correlated with all five mindfulness scales used here for validation purposes (MAAS, FMI, CAMS-R, and SMQ), but correlations were generally weaker for TMS Curiosity (*r* = .22 to .48) than for TMS Decenter (*r* = .47 to .74) [39]. In the development study of the PHLMS [40], the subscales correlated weakly to moderately with the MAAS.

Finally, the factor structure of the FFMQ was examined individually and jointly with the PHLMS and TMS subscales, in order to further examine the validity of the respective facets (or subscales) as indicators of mindfulness and how to best organise them structurally. In keeping with previous findings and the problematic pattern of associations exhibited by FFMQ Observe, the four-factor hierarchical model omitting this facet fit the data best in both samples. However, facet loadings were heterogeneous, with two facets (Describe and Nonreact) showing markedly weaker loadings on the latent mindfulness factor. As observed previously [18], the two facets with the strongest loadings in both samples were Act with Awareness and Accept without Judgment. A joint Exploratory Factor Analysis on item parcels of these four FFMQ facets, combined with item parcels of the PHLMS and TMS subscales, showed that PLHMS Acceptance indeed overlaps with FFMQ Accept without Judgment, although a follow-up analysis indicated that these two variables are also distinct. Indeed, Confirmatory Factor Analysis identified PHLMS Acceptance as an independent factor under the global mindfulness construct, along with the four remaining FFMQ facets.

### Implications

The results concerning the FFMQ, and in particular its Observe facet, fit into an increasingly observed pattern of findings that speaks to the distinctiveness of this facet. Provided that the other four FFMQ facets represent mindfulness, it would not be unreasonable to drop the Observe facet entirely in non-meditating samples. The problem is that such facets compromise the validity and explanatory effects of the global composite and measure when combined with the other facets [46].

The findings confirm that the two subscales of both multidimensional measures scrutinised in this study assess distinct dimensions that either overlap to a small degree (TMS) or are completely orthogonal (PHLMS). Of particular interest, however, is that the variance of at least one subscale of each measure seems largely accounted for by a different construct than that underlying the unidimensional facet or item mindfulness scales. Specifically, the shared variance of PHLMS Awareness and especially of TMS Curiosity with mindfulness appears to be negligible; both had insufficient loadings on, and associations with, mindfulness to be considered useful facets of the global construct. Therefore, the findings do not support the validity of these two subscales as indicators of mindfulness. In fairness, it is important to emphasise that neither of them is conceptualised as representing a global mindfulness factor, although each has been linked to the concept. Together with the LMS, which is also dimensionally distinct from the mainstream conceptualisation of mindfulness, use of the TMS Curiosity and PHLMS Awareness subscales for the purpose of assessing “mindfulness” is not empirically supported.

In contrast, the findings show that the PHLMS Acceptance subscale represents a distinct facet of the mainstream mindfulness conceptualisation. In particular, it seems to measure a facet akin to FFMQ Accept without Judgment. However, its associations with this corresponding FFMQ facet was not large enough to suggest equivalence, or that it measures the same attribute to a similar degree. It is possible that this subscale represents the facet partially but also incorporates manifestations of the facet not already covered in the FFMQ or similar measures.

The findings pertaining to PHLMS Awareness, and to a lesser extent TMS Curiosity, may have further key implications for the representation and measurement of mindfulness. As discussed, the PHLMS assesses Awareness orthogonally to Acceptance, whereas the conceptually similar FFMQ/KIMS Observe facet is treated obliquely to the other facets, including Acceptance. Yet, the present findings show that PHLMS Awareness (and not just Acceptance) also correlates well with its corresponding FFMQ facet (Observe). Since research is increasingly identifying the FFMQ/KIMS Observe facet as problematic (i.e., as distinct from the mindfulness factor related to the other facets), [40] may be on the right track in assessing Awareness as a distinct dimension (from Acceptance). The similar concepts reflected in PHLMS Awareness, FFMQ/KIMS Observe, and TMS Curiosity appear to be largely different from the mindfulness dimension underlying most of the existing measures.

### Limitations and Future Directions

Although the results were generally similar across the two samples, it is important to note that the Sample 2 data were collected online with relatively little control over who completed the survey and how. The Sample 1 data were also collected online, but these participants were recruited via the participant pool of a university, which imposes a greater degree of control and participation etiquette. Even though the data were rigorously pre-screened to identify problem responses, some invalid or poor quality responses may always go unnoticed. At the same time, the use of two (very different) samples is a strength of the study, with consistency in results strengthening the inferences made.

A related limitation is the use of convenience samples, with uneven distributions of demographic factors (e.g., gender) possibly impinging on the pattern of results obtained here. Although this study focused on the common pattern of associations between the two samples, it is worth noting that, in general, the correlations appeared to be somewhat larger in the online sample. Demographic factors, which were beyond the scope here, warrant greater attention in future research, especially in light of mixed evidence for measurement invariance [47,48]. A related limitation was that no data on meditation experience was collected, in part because most of the findings of measurement variance between meditators and non-meditators have only been published recently, following the data collection. Since neither of them were specifically selected from the meditating population, it must be tentatively assumed that they comprised mostly non-meditators, rendering the findings mostly valid for this group. Research scrutinising the psychometric differences of meditators more systematically is warranted, given the close tie of meditation and mindfulness.

The present study showed that certain elements of the PHLMS and TMS seem to qualify as facets of a global mindfulness construct. A next logical step would be to systematically examine if these subscales have any added representational value, or even advantage, relative to the FFMQ facets. PHLMS Acceptance may occupy unique construct variance not already covered by conceptually similar or equivalent FFMQ facets. For example, PHLMS Acceptance and FFMQ Accept without Judgment could be compared in their capacity to predict specific behaviours or states relevant to mindfulness. Simultaneous comparison of facets and subscales between the various measures can be very informative in regards to optimising the representation and operationalisations of mindfulness.

## Compliance with Ethical Standards

All procedures performed in studies involving human participants were in accordance with the ethical standards of the institutional and/or national research committee and with the 1964 Helsinki declaration and its later amendments or comparable ethical standards.

Informed consent was obtained from all individual participants included in the study.

## Supporting Information

S1 Data Complete Data File. (ZIP)

S1 Table Pattern Matrix for Promax Eight-Factor Solution Extracted from FFMQ, TMS, and PHLMS Item Parcels Corresponding to Each Facet or Subscale and Factor Correlation Matrix in Sample 2. (DOCX)
