# Supplementary material for: Zeroing In on Mindfulness Facets: Similarities, Validity, and Dimensionality across Three Independent Measures
Source: PLoS One. 2016 Apr 7;11(4):e0153073. doi: 10.1371/journal.pone.0153073 (PMC4824528; doi:10.1371/journal.pone.0153073)
Supplement: S1 Table — (DOCX) [file pone.0153073.s002.docx]

**S1 Table. Pattern Matrix for Promax Eight-Factor Solution Extracted from FFMQ, TMS, and PHLMS Item Parcels Corresponding to Each Facet or Subscale and Factor Correlation Matrix in Sample 2**

| **Parcel** | **Factor loading** | | | | | | | |
| --- | --- | --- | --- | --- | --- | --- | --- | --- |
|  | **1** | **2** | **3** | **4** | **5** | **6** | **7** | **8** |
| FFMQ AWA P1 | .915 |  |  |  |  |  |  |  |
| FFMQ AWA P3 | .871 |  |  |  |  |  |  |  |
| FFMQ AWA P2 | .831 |  |  |  |  |  |  |  |
| FFMQ AWJ P1 |  | .897 |  |  |  |  |  |  |
| FFMQ AWJ P2 |  | .882 |  |  |  |  |  |  |
| FFMQ AWJ P3 |  | .826 |  |  |  |  |  |  |
| FFMQ Describe P1 |  |  | .896 |  |  |  |  |  |
| FFMQ Describe P2 |  |  | .872 |  |  |  |  |  |
| FFMQ Describe P3 |  |  | .792 |  |  |  |  |  |
| TMS Curiosity P2 |  |  |  | .947 |  |  |  |  |
| TMS Curiosity P3 |  |  |  | .898 |  |  |  |  |
| TMS Curiosity P1 |  |  |  | .641 |  |  |  |  |
| FFMQ Nonreact P1 |  |  |  |  | .823 |  |  |  |
| FFMQ Nonreact P3 |  |  |  |  | .818 |  |  |  |
| FFMQ Nonreact P2 |  |  |  |  | .732 |  |  |  |
| PHLMS Acceptance P3 |  |  |  |  |  | .833 |  |  |
| PHLMS Acceptance P1 |  |  |  |  |  | .741 |  |  |
| PHLMS Acceptance P2 |  |  |  |  |  | .699 |  |  |
| PHLMS Awareness P1 |  |  |  |  |  |  | .771 |  |
| PHLMS Awareness P3 |  |  |  |  |  |  | .760 |  |
| PHLMS Awareness P2 |  |  |  |  |  |  | .700 |  |
| TMS Decenter P3 |  |  |  |  |  |  |  | .880 |
| TMS Decenter P2 |  |  |  |  |  |  |  | .572 |
| TMS Decenter P1 |  |  |  |  |  |  |  | .547 |

*N* = 172. Factor loadings of < .30 are omitted from the table. FFMQ = Five Facet Mindfulness Questionnaire [18]; PHLMS = Philadelphia Mindfulness Scale [40]; TMS = Toronto Mindfulness Scale [39]; AWJ = Accept w/o Judgment; AWA = Act with Awareness; P1 to P3 = Parcels 1 to 3.
